# Maternal Parity and the Risk of Congenital Heart Defects in Offspring: A Dose-Response Meta-Analysis of Epidemiological Observational Studies

**DOI:** 10.1371/journal.pone.0108944

**Published:** 2014-10-08

**Authors:** Yu Feng, Di Yu, Tao Chen, Jin Liu, Xing Tong, Lei Yang, Min Da, Shutong Shen, Changfeng Fan, Song Wang, Xuming Mo

**Affiliations:** 1 Department of Cardiothoracic Surgery, The Affiliated Children's Hospital of Nanjing Medical University, Nanjing, Jiangsu, China; 2 Department of Epidemiology and Biostatistics, School of Public Health, Nanjing Medical University, Nanjing, Jiangsu, China; 3 Atherosclerosis Research Center, Key Laboratory of Cardiovascular Disease and Molecular Intervention, Nanjing Medical University, Nanjing, Jiangsu, China; 4 Department of Cardiology, The First Affiliated Hospital of Nanjing Medical University, Nanjing, Jiangsu, China; Bambino Gesù Children's Hospital, Italy

## Abstract

**Background:**

Epidemiological studies have reported conflicting results regarding maternal parity and the risk of congenital heart defects (CHDs). However, a meta-analysis of the association between maternal parity and CHDs in offspring has not been conducted.

**Methods:**

We searched MEDLINE and EMBASE for articles catalogued between their inception and March 8, 2014; we identified relevant published studies that assessed the association between maternal parity and CHD risk. Two authors independently assessed the eligibility of the retrieved articles and extracted data from them. Study-specific relative risk estimates were pooled by random-effects or fixed-effects models. From the 11272 references, a total of 16 case-control studies and 3 cohort studies were enrolled in this meta-analysis.

**Results:**

The overall relative risk of CHD in parous versus nulliparous women was 1.01 (95% CI, 0.97–1.06; *Q* = 32.34; *P* = 0.006; *I^2^* = 53.6%). Furthermore, we observed a significant association between the highest versus lowest parity number, with an overall RR = 1.20 (95% CI, 1.10–1.31; (*Q* = 74.61, *P*<0.001, *I^2^* = 82.6%). A dose–response analysis also indicated a positive effect of maternal parity on CHD risk, and the overall increase in relative risk per one live birth was 1.06 (95% CI, 1.02–1.09); *Q* = 68.09; *P*<0.001; *I^2^* = 80.9%). We conducted stratified and meta-regression analyses to identify the origin of the heterogeneity among studies. A Galbraith plot was created to graphically assess the sources of heterogeneity.

**Conclusion:**

In summary, this meta-analysis provided a robust estimate of the positive association between maternal parity and risk of CHD.

## Introduction

Congenital heart defects (CHD) are the most common human birth defects and the leading cause of perinatal mortality, with an incidence of approximately 4 to 50 per 1000 live birth or even higher [1]. The etiology of CHD is complex and may involve the interaction of environmental exposure and inherited factors [2]. A multitude of studies have identified both chromosomal and gene mutations as the cause of the syndromic version of the heart malfunction [1]. In contrast, the origin of non-syndromic CHD, which accounts for most congenital cardiac abnormalities, remains unknown.

Maternal phenylketonuria, diabetes mellitus, maternal teratogen exposure, and maternal therapeutic drug exposure during pregnancy may increase the risk of congenital malformations in offspring [3]. Apart from these influences, previous studies have indicated that inherent maternal characteristics, such as parity, may be responsible for certain categories of congenital defects. Some studies have observed a positive association between nulliparity and the risk of various birth defects [4]–[10]. In contrast, other studies have observed that multiparity is associated with an increased risk of specific birth defects [11]–[13]. The results for CHD are similar; no consensus has been reached, and some studies show positive associations while others find null results. The association between maternal parity and CHDs might be explained by unmeasured environmental risk factors which are more common among multiparous women than nulliparous women. Both biological and psychosocial interpretations can be proposed, including maternal stress, maternal uterus condition and serum levels of estradiol [14]–[17].

To date, an increasing number of studies has focused on the association between maternal parity and CHDs; however, the results have been ambiguous, possibly because of inadequate sample sizes. Therefore, we conducted a dose-response meta-analysis to quantitatively assess the effects of maternal parity on CHDs.

## Methods

### Literature Search

To identify relevant epidemiological studies, two independent researchers (Feng and Yu) conducted a computerized literature search in MEDLINE and EMBASE to retrieve articles that were catalogued between the databases' inception and March 8, 2014.The search terms for the exposure were: ‘Parity’, ‘Pregnancy’, ‘Live Birth’, ‘Reproduction’, ‘Reproductive’ and ‘Reproductive Factors’ and the search terms for the outcome were: ‘Congenital Heart Defect’, ‘Heart Abnormality’, ‘Malformation Of Heart’ and ‘CHD’. In addition, we conducted a search for a broad range of environmental teratogens and CHDs and examined the relevant references and review articles; in this way, we could identify information from other related studies. We followed standards of quality for conducting and reporting meta-analyses [18].

### Eligibility Criteria

We selected articles that (1) were original epidemiologic studies (i.e., case–control and cohort), (2) examined the association between maternal parity and CHDs overall or any one of the CHD subtypes in infants, (3) were published in the English language, (4) reported RRs (i.e., risk ratios or odds ratios) and associated 95% confidence intervals (CIs) or standard errors or provided the data necessary to recalculate these factors, and (5) defined CHDs or one of the CHD subtypes as an outcome. Articles that reported results from more than one population were considered to be separate studies. When multiple articles from the same study were provided, we used the article with the most applicable information and the largest number of cases. We excluded non-peer-reviewed articles, experimental animal studies, ecological assessments, correlation studies and mechanistic studies.

### Data Extraction

Data extraction was carried out separately by two reviewers (Feng and Yu) working independently. When differences of opinion arose, they were resolved by a discussion between the two reviewers or by the involvement of a third reviewer (Chen) for adjudication. Parity was defined as the number of live births before the index delivery [19]. Nulliparous women were defined as those with no previous live births before the index delivery. Primiparous women were those with one live birth, and multiparous women were those with two or more prior live births. The studies that met the inclusion criteria were reviewed to retrieve the information of interest. The characteristics of interest included authors, year of publication, geographic region, periods of data collection, study design, sample size, case classification, exposure and outcome assessment (including parity as both a binary and categorical variable), adjusted estimates and their corresponding 95% CIs for parous versus nulliparous women, highest versus lowest number of previous births, and confounding factors that were controlled for by matching cases or adjustments in the data analysis. We back-calculated the point estimate and 95% CI if the original study did not report the risk estimates in this order. When no adjusted estimates were available, we extracted the crude estimate. If no estimate was provided in a given study, we recalculated odds ratios or risk ratios and 95% CIs from the presented raw data using standard equations.

To assess the study quality, we used a 9-star system on the basis of the Newcastle-Ottawa Scale [20]. This system judges a study based on three broad characteristics: the selection of study groups, comparability of study groups and ascertainment of the exposure or outcome of interest for case-control and cohort studies, respectively. The highest score was 9, and we defined a high quality study as one with a quality score greater than or equal to 7.

### Statistical Analysis

We used study-specific relative risks as a summary statistic of the association between maternal parity and CHD risk. To simplify the procedure, a RR was used to represent all reported study-specific results from cohort studies and an OR to represent results from case-control studies. If a study did not use the lowest parity number as the reference category, the effective count method proposed by Hamling and colleagues [21] was used to recalculate the RRs.

For the dose–response analysis, which considers parity as a continuous variable, the method proposed by Greenland and colleagues [22] and Orsini and colleagues [23] was used to calculate study-specific slopes (i.e., linear trends) and 95% CIs. For studies which reported duration as a range, the midpoint, determined by calculating the average of the lower and upper bounds, was used. When the highest category was open-ended, the width of the open-ended interval was taken to be the same as that of the category immediately previous to it. When the lowest category did not have a lower bound, we considered the lower bound to be zero. We presented the dose–response results in forest plots on the basis of increments of 1 live birth with regard to parity.

Cochran's *Q* and *I^2^* statistics were used to test for heterogeneity among studies [24]. If there was evidence of heterogeneity (*P*<0.05 or *I^2^*≧56%), a random-effects model was used, which provided a more appropriate summary estimate for heterogeneous study-specific estimates. If the study revealed no evidence of heterogeneity, the fixed-effects analysis was used, an inverse variance weighting was applied to calculate summary RR estimates [25].

We conducted subgroup analyses based on study design (i.e., cohort versus case–control studies), geographical region (i.e., North America, Europe, and Asia), number of cases (i.e., ≤1000 versus >1000), publication period (i.e., before 2010 versus 2010 or after), maternal age (i.e., ≤27 versus >27), primary interest (i.e., whether the title or abstract refers to the reproductive factors as their research interest, yes versus no), and study quality (i.e., low versus high quality). We evaluated heterogeneity between subgroups by meta-regression. A *P* value less than 0.05 from the meta-regression was considered representative of a significant difference between subgroups. Finally, we conducted sensitivity analyses to explore whether a specific study strongly influenced the results by excluding one study at a time.

Publication bias was assessed via visual inspection of a funnel plot with asymmetry using both Egger's linear regression [26] and Begg's rank correlation [27] methods. Significant statistical publication bias was defined as a *P* value of <0.05 for the two above-mentioned tests. All statistical analyses were performed with STATA (version 11.0; StataCorp, College Station, Texas, USA).

## Results

### Study Characteristics

**Figure 1:**
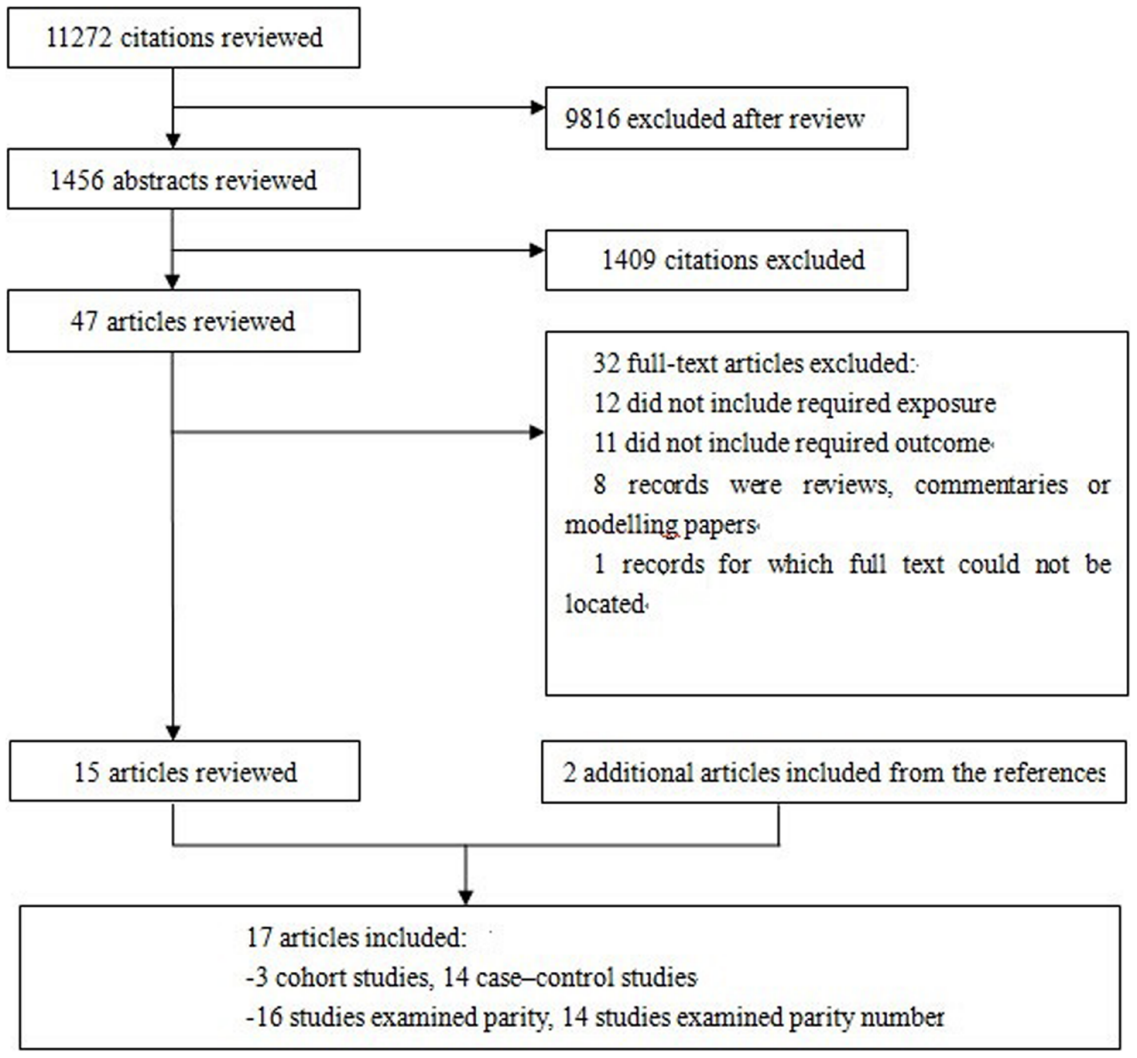
Study selection procedures for a meta-analysis of maternal parity and congenital heart defects (CHDs) in offspring.

The search strategy generated 11272 citations; from these, 17 were used in the final analysis, representing 43880 incident cases (Figure 1). All of the studies were published between 1989 and 2013. There were 14 case–control studies [28]–[41] and 3 cohort studies [42]–[44]. The main characteristics of the included studies are presented in Table S1. As shown, 10 studies [28]–[30], [32], [33], [35], [36], [39], [43], [44] were conducted in the United States or Canada, 6 in Europe [31], [37], [38], [40]–[42], and 1 in Asia [34]. Among these studies, 16 investigated the association between maternal parity as a binary variable and CHD risk [28]–[43], and 14 examined the association of maternal parity number with CHD risk [28], [30]–[34], [36], [38]–[44]. In the 3 cohort studies, cohort sizes varied from 22,365 [44] to 1,625,945 [43], and the number of CHD cases ranged from 4,123 [44] to 12,101 [43]. In the 16 case–control studies, the number of cases varied from 81 [28] to 7,575 [32], and the number of control subjects ranged from 302 [39] to 38,151 [41]. The highest parity number ranged from 2 [28] to more than 4 [39].

### Parous versus Nulliparous

**Figure 2:**
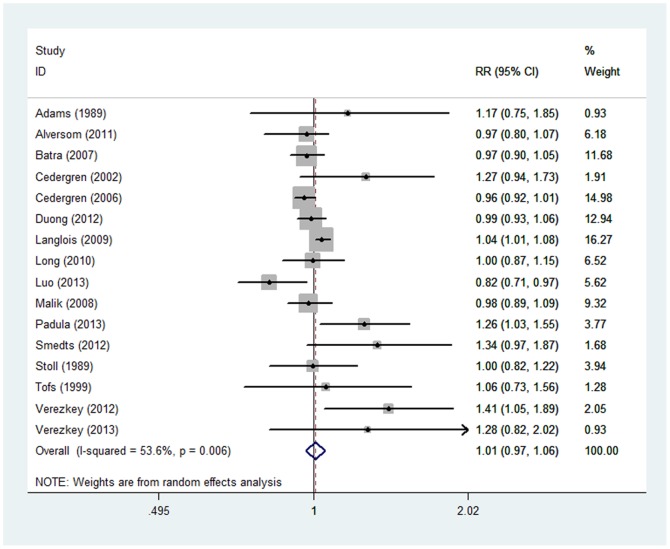
Relative risk (RR) estimates for the association between ever parity and CHD risk. Meta-analysis random-effects estimates were used. The sizes of the squares reflect the weighting of the included studies. Bars represent 95% confidence intervals (CIs). The center of the diamond represents the summary effect; left and right points of the diamond represent the 95% confidence interval.

A total of 14 case–control studies and 2 cohort studies examined the association between parity as a binary variable and CHD risk. The overall relative risk of CHD for parous versus nulliparous women was 1.01 (95% CI, 0.97–1.06), with moderate heterogeneity (*Q* = 32.34; P = 0.006; *I^2^* = 53.6%; **Table 1** and **Fig. 2**). There was no indication of publication bias based on the Egger test (*P* = 0.295) or visual inspection of the funnel plot (data not shown). In a sensitivity analysis, we sequentially excluded one study at a time and reanalyzed the data. The 16 study-specific relative risks for the parous versus nulliparous women ranged from a low of 1.01 (95% CI, 0.97–1.05; *Q* = 34.59; *P* = 0.007; *I^2^* = 50.9%) after omission of the study by Padula and colleagues [36] to a high of 1.02 (95% CI, 0.99–1.06; *Q* = 31.44; *P* = 0.018; *I^2^* = 45.9%) after omission of the study by Luo and colleagues [34]. As shown in **Table 1**, similar risks were observed between subgroup stratified by maternal age for association between maternal ever parity and CHD in offspring (*P* for heterogeneity = 0.12).

**Table 1:** Summary risk estimates of the association between maternal ever parity and CHD risk in offspring.

Subgroup analysis	No. of studies	No. of cases	Summary RR (95% CIs)	*P* 1	*I* *^2^* (%)	*P* 2
Summary pooled estimate	16	39757	1.01(0.97–1.06)	0.006	53.6	
Geographical region						0.202
North America	9	31090	1.01(0.98–1.05)	0.313	14.5	
Europe	6	7974	1.14(0.98–1.33)	0.014	64.9	
Asia	1	693	0.82(0.71–0.97)	-	-	
Number of cases						0.438
≤1000	9	3691	1.14(0.98–1.32)	0.007	62.3	
>1000	7	36066	1.00(0.98–1.03)	0.165	34.5	
Publication period						0.719
Before 2010	8	26457	1.00(0.96–1.04)	0.119	39.1	
2010 or after	8	13300	1.05(0.95–1.17)	0.004	66.4	
Design						0.744
Case-control	14	20747	1.02(0.96–1.09)	0.027	46.9	
Cohort	2	19010	1.00(0.93–1.08)	0.006	86.6	
Maternal age(year)						0.12
≤27	8	18296	1.05(0.99–1.12)	0.169	32.4	
>27	7	21461	0.98(0.92–1.04)	0.063	49.8	
Primary interest						0.69
Yes	9	23805	1.01(0.94–1.09)	0.020	54.2	
No	7	15952	1.03(1.00–1.06)	0.144	39.2	
Quality assessment						0.362
High quality studies (scores≥7)	11	30300	1.03(0.99–1.07)	0.060	43.6	
Low quality studies (scores<7)	7	9457	0.96(0.92–1.00)	0.161	36.8	

1 p-value for heterogeneity within each subgroup.

2 p-value for heterogeneity between subgroups with meta-regression analysis.

Abbreviations: RR: relative risk; CI: confidence interval.

### Highest versus Lowest Parity Number

**Figure 3:**
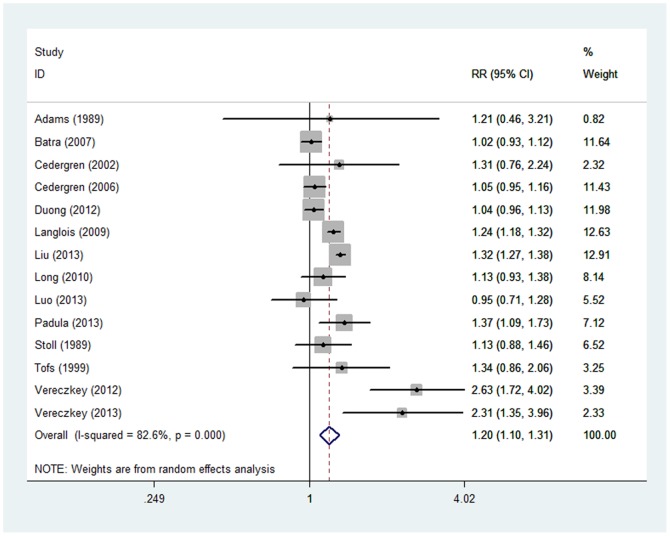
Relative risk (RR) estimates for the association between parity number (highest versus lowest) and CHD risk. Meta-analysis random-effects estimates were used. The sizes of the squares reflect the weighting of the included studies. Bars represent 95% confidence intervals (CIs). The center of the diamond represents the summary effect; left and right points of the diamond represent the 95% confidence interval.

A total of 11 case–control studies and 3 cohort studies examined the association between high and low parity and CHD risk. The estimate of the relative risk of CHD for the highest versus lowest parity categories was 1.20 (95% CI, 1.10–1.31). Statistically significant heterogeneity was detected (*Q* = 74.61, *P*<0.001, *I^2^* = 82.6%; Table 2 and Fig. 3) with no publication bias (Begg's test: *P* = 0.443, Egger's test: *P* = 0.883). The 13 study-specific relative risks when considering the parity number ranged from a low of 1.17 (95% CI, 1.07–1.27; *Q* = 61.84; *P* = 0.000; *I^2^* = 80.6%) after omission of the study by Vereczkey and colleagues [40] to a high of 1.22 (95% CI, 1.12–1.34; *Q* = 59.74; *P* = 0.000; *I^2^* = 79.9%) after omission of the study by Batra and colleagues [30].

**Table 2:** Summary risk estimates of the association between maternal parity number and CHD risk in offspring.

	Highest versus lowest						Dose–response analysis (per 1 live birth)					
Subgroup analysis	No. of studies	No. of cases	Summary RR (95% CIs)	*P* 1	*I* *^2^*(%)	*P* 2	No. of studies	No. of cases	Summary RR (95% CIs)	*P* 1	*I* *^2^*(%)	*P* 2
Summary pooled estimate	14	38027	1.21(1.11–1.31)	<0.001	83.8		14	38027	1.06(1.02–1.09)	<0.001	80.9	
Geographical region						0.924						0.379
North America	8	29621	1.18(1.07–1.30)	<0.001	84.3		8	29621	1.05(1.02–1.08)	<0.001	75	
Europe	5	7713	1.49(1.06–2.09)	<0.001	83.6		5	7713	1.17(1.02–1.34)	<0.001	83.1	
Asia	1	693	0.95(0.71–1.28)	-	-		1	693	0.92(0.82–1.04)			
Number of cases						0.140						0.060
≤1000	8	3430	1.41(1.12–1.79)	<0.001	66.1		8	3430	1.14(1.03–1.25)	<0.001	71.6	
>1000	6	34597	1.13(1.03–1.25)	<0.001	90.4		6	34597	1.04(1.00–1.07)	<0.001	88	
Publication period						0.340						0.665
Before 2010	7	23390	1.13(1.02–1.25)	<0.001	65.2		7	23390	1.03(1.02–1.05)	0.110	42	
2010 or after	7	14637	1.30(1.12–1.53)	<0.001	87.4		7	14637	1.09(1.02–1.16)	<0.001	82.7	
Design						0.829						0.626
Case-control	11	15539	1.22(1.07–1.39)	<0.001	70.1		11	15539	1.05(0.97–1.13)	<0.001	96.4	
Cohort	3	22488	1.21(1.09–1.34)	<0.001	88.8		3	22488	1.05(1.01–1.10)	<0.001	93.5	
Maternal age(year)						0.106						0.157
≤27	7	15229	1.37(1.16–1.63)	0.004	68.6		7	15229	1.12(1.04–1.20)	0.001	72.5	
>27	7	22798	1.11(0.97–1.27)	<0.001	88.8		7	22798	1.03(0.99–1.08)	<0.001	86.9	
Primary interest						0.774						0.737
Yes	10	21534	1.23(1.08–1.40)	<0.001	84.7		10	21534	1.06(1.02–1.11)	<0.001	84.1	
No	4	16493	1.18(1.01–1.37)	0.003	78.6		4	16493	1.05(1.00–1.10)	0.049	61.9	
Quality assessment						0.252						0.673
High quality studies (scores≥7)	8	28570	1.27(1.13–1.43)	0.000	88.6		8	28570	1.08(1.03–1.12)	0.000	83.6	
Low quality studies (scores<7)	6	9457	1.08(0.99–1.16)	0.735	0		6	9457	1.01(1.00–1.04)	0.251	24.4	

1 p-value for heterogeneity within each subgroup.

2 p-value for heterogeneity between subgroups with meta-regression analysis.

Abbreviations: RR: relative risk; CI: confidence interval.

### Dose–Response Analysis

**Figure 4:**
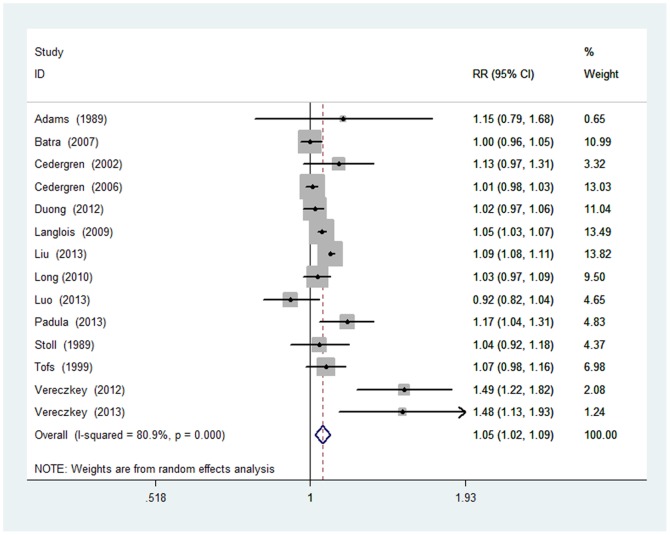
Relative risk (RR) estimates for the association between parity number (per 1 live birth) and CHD risk. Meta-analysis random-effects estimates were used. The sizes of the squares reflect the weighting of the included studies. Bars represent 95% confidence intervals (CIs). The center of the diamond represents the summary effect; left and right points of the diamond represent the 95% confidence interval.

A total of 11 case–control studies and 3 cohort studies were included in the dose-response analysis. The estimate of relative risk per live birth was 1.06 (95% CI, 1.02–1.09), and there was statistically significant heterogeneity (*Q* = 68.09; *P*<0.001; *I^2^* = 80.9%; Table 2 and Fig. 4). Publication bias was not evident based on the Egger test (*P* = 0.973) or Begg test (*P* = 0.101), and no asymmetry was observed in the funnel plots. The 13 study-specific relative risks of parity ranged from a low of 1.05 (95% CI, 1.02–1.08; *Q* = 56.94; *P* = 0.000; *I^2^* = 78.9%) after omission of the study by Vereczkey and colleagues [40] to a high of 1.06 (95% CI, 1.03–1.10; *Q* = 51.12; *P* = 0.000; *I^2^* = 76.5%) after omission of the study by Cedergren and colleagues [42].

### Heterogeneity Analysis

We conducted stratified and meta-regression analyses to identify the origin of the heterogeneity among studies. In subgroup analyses of parity as a binary variable and CHD risk, there was no indication of significant heterogeneity between subgroups according to meta-regression analyses (**Table 1**). However, significant heterogeneity existed in the dose-response analyses of the association between parity number and CHD risk. To clarify the sources of heterogeneity, we conducted a sensitivity analysis; however, *I^2^* did not decrease much by removing each study in turn. Subsequently, a meta-regression was performed with a Knapp-Hartung modification, and we found that differing numbers of cases may contribute to the heterogeneity (p = 0.060). We further created a Galbraith plot to graphically assess the sources of heterogeneity (Figures S1, S2). A total of 7 studies [30], [32], [34], [40]–[42], [44] were identified as the primary sources of heterogeneity (i.e., 6 studies [30], [32], [40]–[42], [44] from the high versus low parity number analysis and 6 studies [30], [34], [40]–[42], [44] from the dose-response analysis). Once the outlying studies were excluded, the heterogeneity was effectively removed (i.e., for the high versus low parity number analysis, *I^2^* = 0.0%; for the dose-response analysis, *I^2^* = 0.0%); however, the corresponding pooled RRs were not materially altered in any comparisons (i.e., for the high versus low parity analysis: RR = 1.23, 95% CI = 1.17–1.29; for the dose-response analysis: RR = 1.05, 95% CI = 1.03–1.07).

## Discussion

To the best of our knowledge, this is the first quantitative meta-analysis evaluating the association between maternal parity and the risk of congenital heart defects. Overall, the findings of our meta-analysis suggested that maternal parity (i.e., the highest category compared to the lowest category, RR = 1.20, 95% CI = 1.10–1.31) was significantly associated with CHD risk. Meanwhile, in the dose-response meta-analysis, we found that the risk of CHD increased by 6% per live birth. However, there was no evidence that verified the association between parous versus nulliparous women (RR = 1.01, 95%CI = 0.97–1.06) and the risk of CHDs. Additionally, the results were consistent across most of the subgroup analyses (Table 1 and 2).

Although the specific biological mechanism underlying maternal parity and the risk of CHDs remains unclear, some relevant evidence has been published. Nutrient depletion was more likely to occur among mothers who had given birth to live fetuses than those who had never delivered. Folic acid is one of the most important vitamins, and the association between folic acid and birth defects has been widely studied. It has been confirmed that lack of it would cause severe congenital malformation [45], especially CHDs [46] and neural tube defects [47]. Additionally, mothers who gave birth to more fetuses were more likely to have shorter inter-pregnancy intervals, which have been verified to increase the risk of major congenital malformations, including CHDs [48]. Moreover, having young children who carry respiratory viruses in the household would increase the risk of an embryo's in utero exposure to viruses, such as rubella, which was confirmed to contribute to CHD more than half a century ago [49], [50]. Moreover, changes in the intrauterine environment that affect embryonic development and eventually lead to birth defects may be explained by multiparity. In addition to biological interpretations, psychosocial explanations should also be explored. Multiparity would cause an increased burden on families and increased mental stress in parents. Moreover, Zhu et al [14] found that mothers who were exposed to stress during pregnancy were at an increased risk of having offspring with CHD.

When stratified by geographic region, a significant increase in CHD risk in North America and Europe was found to be associated with increases in parity number, and similar results were found in a dose-response meta-analysis. However, the pooled RRs for North America and Europe differed when considering parity as a binary variable. Considering the fact that only one study from Asia was included, the influence of parity in this region needs further research. In the subgroup analysis to assess study quality, we observed statistically significant results in high quality studies that included analyses of both parity number and dose-response, while no significant association was found among low quality studies. For the subgroup analysis of study design, the pooled RR from case-control studies was different from cohort studies in the analysis of dose-response. Selection and information biases might account for the observed difference. Furthermore, compared to the cohort studies, the case-control studies had a lower median quality score (7 versus 8), which may have an influence on the results.

Some limitations of our study must be taken into account. First, a total of 14 case-control studies and 3 cohort studies were recruited into our meta-analysis, and we extracted our raw data primarily from case-control studies, which are susceptible to selection and information biases. Additionally, our meta-analysis was limited to studies published in English; the results may therefore have been affected by the lack of data from studies performed in other languages. Thus, general conclusions must be considered carefully and cannot be regarded as the final word on the matter. Second because we lacked a large data set, we did not conduct a subgroup analysis of CHD subtypes; however, different CHD subtypes have different etiologies. Maternal parity may be not associated with all subtypes of CHD. Therefore, further research, including more high quality studies, is needed. Thirdly, although no evidence of publication bias was found, heterogeneity exists among the studies included in these analyses of both parity number and dose response; this heterogeneity may affect the interpretation of the overall results. In this study, we conducted sensitivity analyses to explore the sources of heterogeneity by deleting one study at a time from the pooled analysis. However, heterogeneity still could not be fully removed. Moreover, geographical region, sample size, CHD subtypes and other risk factors may result in heterogeneity. Therefore, we performed meta-regression and subgroup analyses to further investigate the sources of heterogeneity. In the dose-response analysis, we found that the heterogeneity stemmed partly from the number of cases. In contrast, no cause was found for the heterogeneity in the parity number meta-analysis. Furthermore, we created a Galbraith plot to assess the heterogeneity and to identify potentially outlying studies. A total of 6 were identified as the primary contributors to heterogeneity in both the analysis of parity number [30], [32], [40]–[42], [44] and dose-response [30], [34], [40]–[42], [44]. After excluding the outlying studies, the above-mentioned heterogeneity was effectively removed while the corresponding pooled RRs were not materially altered, indicating that the overall results regarding parity number and dose-response were statistically stable. Meanwhile, in the subgroup analysis to assess quality, heterogeneity was present in the high quality studies but not in the low quality ones. Of the 7 studies that were the main sources of heterogeneity, 5 [31], [33], [41], [42], [45] were high quality studies, which could explain the discrepancy. Finally, maternal age may be a major confounder, but in our study, similar risks were observed between subgroup stratified by maternal age for association between maternal parity and CHD in offspring (*P* for heterogeneity = 0.12 in maternal ever parity; *P* for heterogeneity = 0.106 and *P* for heterogeneity = 0.157 in maternal parity number). So we consider that maternal age may have no significant confounding effect on association between maternal parity and CHD in offspring. However, because of the limiting number of included studies, more studies are needed to validate our results.

Additionally, there are several important strengths of our study. First, to our knowledge, this is the first meta-analysis to report an association between maternal parity and CHDs. Moreover, our literature search was conducted on multiple databases, and the references from the retrieved articles were fully scrutinized to obtain any missing data. Therefore, our study included 43880 cases, enough to have sufficient statistical power to investigate the potential association between maternal parity and the risk of CHDs. Another strength of our study is that, although heterogeneity exists in our meta-analysis, we conducted a number of sensitivity, subgroup and Galbraith plot analyses and found that the results were stable.

In summary, this study provides evidence that maternal parity number was positively associated with the risk of CHDs. However, more prospective studies, particularly in developing countries, are needed to further investigate the association between maternal parity and CHDs, especially with regard to the different subtypes of CHDs.

## Supporting Information

Figure S1 **Galbraith plots for parity number (highest versus lowest) and CHD risk.**
(TIF)

Figure S2 **Galbraith plots for parity number (per 1 live birth) and CHD risk.**
(TIF)

Table S1 **Characteristics of studies of maternal parity and CHD risk.**
(DOC)

Checklist S1 **PRISMA checklist.**
(DOC)
