# Supplementary figures and images for: Maternal Parity and the Risk of Congenital Heart Defects in Offspring: A Dose-Response Meta-Analysis of Epidemiological Observational Studies
Source: PLoS One. 2014 Oct 8;9(10):e108944. doi: 10.1371/journal.pone.0108944 (PMC4189919; doi:10.1371/journal.pone.0108944)

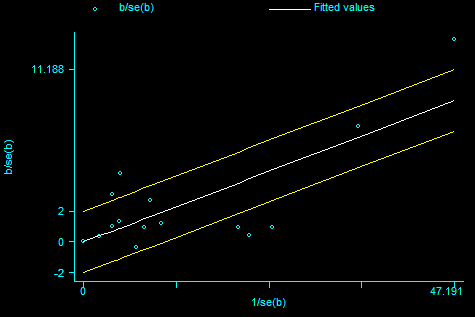

Supplement: Figure S1 — Galbraith plots for parity number (highest versus lowest) and CHD risk. (TIF) [file pone.0108944.s001.tif]

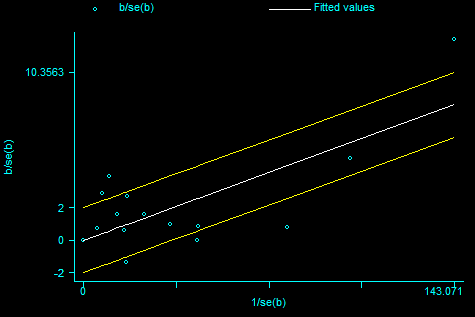

Supplement: Figure S2 — Galbraith plots for parity number (per 1 live birth) and CHD risk. (TIF) [file pone.0108944.s002.tif]
