# Supplementary material for: Maternal Parity and the Risk of Congenital Heart Defects in Offspring: A Dose-Response Meta-Analysis of Epidemiological Observational Studies
Source: PLoS One. 2014 Oct 8;9(10):e108944. doi: 10.1371/journal.pone.0108944 (PMC4189919; doi:10.1371/journal.pone.0108944)
Supplement: Table S1 — Characteristics of studies of maternal parity and CHD risk. (DOC) [file pone.0108944.s003.doc]

|  | Table S1.Characteristics of studies of maternal parity and CHD risk in offspring. | | | | | | | | | | |
| --- | --- | --- | --- | --- | --- | --- | --- | --- | --- | --- | --- |
| First author, year | | Region | Period | Study design/ Source of cases | No. of  cases/ control | Parity categories | No. of  cases/ control | OR/RR | 95%CI | Adjustment  variables^b^ | Outcome |
| Adams, 1989 | | USA | 1976-1980 | Population-based case-control/  The Metropolitan Atlanta Congenital Defects Program | 81/1272 | Nulliparous | 33/569 | 1.00 | Reference | No | CTA |
|  |  |  |  |  |  | 1 | 45/646 | 1.20 | 0.76-1.91 |  |  |
|  |  |  |  |  |  | 2 | 5/71 | 1.21 | 0.46-3.21 |  |  |
|  |  |  |  |  |  | Ever parous | 50/717 | 1.00 | 0.80-1.20 |  |  |
| Alverson, 2011 | | USA | 1987-1988 | Population-based case-control/  The Baltimore- Washington Infant Study (BWIS) | 2525/3435 | Nulliparous | 1246/1668 | 1.00 | Reference | No | CHDs |
|  |  |  |  |  |  | Ever parous | 1279/1767 | 0.97 | 0.80-1.07 |  |  |
| Batra, 2007 | | USA | 1987-2203 | Population-based case-control/ | 3489/13290 | Nulliparous | 1456/5480 | 1.00 | Reference | No | VSD |
|  |  |  |  |  |  | 1 | 1069/4281 | 0.94 | 0.86-1.03 |  |  |
|  |  |  |  |  |  | ≥2 | 892/3297 | 1.02 | 0.93-1.12 |  |  |
| Cedergren, 2002 | | Sweden | 1982-1996 | Population-based case-control /  The Child Cardiology Register | 269/524 | Nulliparous | 96/217 | 1.00 | Reference | No | CHDs |
|  |  |  |  |  |  | 1 | 91/171 | 1.20 | 0.85-1.71 |  |  |
|  |  |  |  |  |  | 2 | 56/91 | 1.39 | 0.92-2.10 |  |  |
|  |  |  |  |  |  | ≥3 | 26/45 | 1.31 | 0.76-2.24 |  |  |
| Cedergren, 2006 | | Sweden | 1992-2001 | Prospective cohort/  The Swedish Medical Birth Registry | 6264/770355 | Nulliparous | 2715/322813 | 1.00 | Reference | No | CHDs |
|  |  |  |  |  |  | 1 | 2201/282407 | 0.93 | 0.88-0.98 |  |  |
|  |  |  |  |  |  | 2 | 986/114667 | 1.02 | 0.95-1.10 |  |  |
|  |  |  |  |  |  | ≥3 | 444/50468 | 1.05 | 0.95-1.16 |  |  |
| Duong, 2012 | | USA | 1997-2007 | Hospital-based case-control/  The National Birth Defects Prevention Study (NBDPS) | 7575/7173 | Nulliparous | 3135/2953 | 1.00 | Reference | No | CHDs |
|  |  |  |  |  |  | 1 | 2397/2377 | 0.95 | 0.88-1.03 |  |  |
|  |  |  |  |  |  | ≥2 | 2043/1843 | 1.04 | 0.96-1.13 |  |  |
|  |  |  |  |  |  | Ever parous | 4440/4220 | 1.51 | 1.20-1.90 |  |  |
| Langlois, 2009 | | USA | 1999-2003 | Retrospective cohort study/  The Texas Birth Defects Registry  (TBDR) | 12101/1625945 | Nulliparous | 2091/619626 | 1.00 | Reference | No | ASD |
|  |  |  |  |  |  | 1 | 1735/495335 | 1.04 | 0.97-1.11 |  |  |
|  |  |  |  |  |  | 2 | 960/274754 | 1.04 | 0.96-1.12 |  |  |
|  |  |  |  |  |  | ≥3 | 722/170397 | 1.26 | 1.16-1.37 |  |  |
|  |  |  |  |  |  | Nulliparous | 2583/619626 | 1.00 | Reference |  | VSD |
|  |  |  |  |  |  | 1 | 2007/495335 | 0.97 | 0.92-1.03 |  |  |
|  |  |  |  |  |  | 2 | 1113/274754 | 0.97 | 0.91-1.04 |  |  |
|  |  |  |  |  |  | ≥3 | 869/170397 | 1.23 | 1.13-1.32 |  |  |
| Liu, 2013 | | Canada | 2002-2010 | Cohort/  The Canadian Institute for Health Information | 4123/22365 | 1 | 5498/- | 1.00 | 1.00-1.00 | No | CHDs |
|  |  |  |  |  |  | 2 | 3137/- | 1.09 | 1.05-1.14 |  |  |
|  |  |  |  |  |  | ≥3 | 3284/- | 1.32 | 1.27-1.38 |  |  |
| Long, 2010 | | USA | 1999-2004 | Population-based case-control/  The Texas Birth Defects Registry (TBDR) | 1045/5225 | Nulliparous | 31/- | 1.00 | Reference | No | TA |
|  |  |  |  |  |  | 1 | 23/- | 0.92 | 0.54-1.58 |  |  |
|  |  |  |  |  |  | 2 | 13/- | 0.92 | 0.48-1.75 |  |  |
|  |  |  |  |  |  | ≥3 | 11/- | 1.22 | 0.61-2.42 |  |  |
|  |  |  |  |  |  | Nulliparous | 161/- | 1.00 | Reference |  | TGA |
|  |  |  |  |  |  | 1 | 147/- | 1.13 | 0.91-1.42 |  |  |
|  |  |  |  |  |  | 2 | 80/- | 1.08 | 0.83-1.42 |  |  |
|  |  |  |  |  |  | ≥3 | 50/- | 1.06 | 0.77-1.46 |  |  |
|  |  |  |  |  |  | Nulliparous | 213/- | 1.00 | Reference |  | TOF |
|  |  |  |  |  |  | 1 | 151/- | 0.88 | 0.71-1.08 |  |  |
|  |  |  |  |  |  | 2 | 91/- | 0.93 | 0.73-1.19 |  |  |
|  |  |  |  |  |  | ≥3 | 73/- | 1.17 | 0.90-1.53 |  |  |
| Luo, 2013 | | China | 2010-2012 | Population-based case-control/  The Shenzhen Maternal and Child Health Management System | 693/11307 | Nulliparous | 390/- | 1.00 | Reference | A, B, C, D, E, F, G, H, I | CHDs |
|  |  |  |  |  |  | 1 | 236/- | 0.84 | 0.71-1.01 |  |  |
|  |  |  |  |  |  | ≥2 | 67/- | 0.95 | 0.71-1.28 |  |  |
| Malik, 2008 | | USA | 1997-2002 | Population-based case-control/  The National Birth Defects Prevention Study | 3067/3947 | Nulliparous | 1213/1568 | 1.00 | Reference | J | CHDs |
|  |  |  |  |  |  | Ever parous | 1540/2060 | 0.98 | 0.89-1.09 |  |  |
| Padula, 2013 | | USA | 1997-2006 | Hospital-based case-control/  The California Birth Defects Monitoring Program | 822/849 | Nulliparous | 263/323 | 1.00 | Reference | No | CHDs |
|  |  |  |  |  |  | 1 | 247/263 | 1.15 | 0.91-1.46 |  |  |
|  |  |  |  |  |  | ≥2 | 304/272 | 1.37 | 1.09-1.73 |  |  |
|  |  |  |  |  |  | Ever parous | 551/535 | 1.50 | 1.10-2.40 |  |  |
| Smedts, 2012 | | Netherlands | 2003 | Population-based case-control/  The Haven study | 261/325 | Nulliparous | 104/153 | 1.00 | Reference | No | CHDs |
|  |  |  |  |  |  | Ever parous | 157/172 | 1.34 | 0.97-1.87 |  |  |
| Stoll, 1989 | | France | 1979-1986 | Hospital-based case-control | 801/801 | Nulliparous | 368/368 | 1.00 | Reference | No | CHDs |
|  |  |  |  |  |  | 1 | 239/262 | 0.91 | 0.73-1.15 |  |  |
|  |  |  |  |  |  | ≥2 | 194/171 | 1.13 | 0.88-1.46 |  |  |
| Tofs, 1999 | | USA | 1991-1993 | Hospital-based case-control/  The California Birth Defects Monitoring Program (CBDMP) | 385/302 | Nulliparous | 73/61 | 1.00 | Reference | No | CHDs |
|  |  |  |  |  |  | 1-3 | 190/159 | 1.06 | 0.72-1.58 |  |  |
|  |  |  |  |  |  | ≥4 | 122/82 | 1.34 | 0.86-2.06 |  |  |
| Vereczkey, 2012 | | Hungary | 1980-1996 | Population-based case-control/  The Hungarian Congenital Abnormality Registry (HCAR) | 302/469 | Nulliparous | 123/231 | 1.00 | Reference | No | LOSD |
|  |  |  |  |  |  | 1 | 109/188 | 1.09 | 0.79-1.50 |  |  |
|  |  |  |  |  |  | ≥2 | 70/50 | 2.63 | 1.72-4.02 |  |  |
| Vereczkey, 2013 | | Hungary | 1980-1996 | Population-based case-control/  The Hungarian Congenital Abnormality Registry (HCAR) | 77/38151 | Nulliparous | 32/18209 | 1.00 | Reference | No | AVCD |
|  |  |  |  |  |  | 1 | 22/14283 | 0.88 | 0.51-1.51 |  |  |
|  |  |  |  |  |  | ≥2 | 23/5659 | 2.31 | 1.35-3.96 |  |  |

CC, case–control study; CHDs, congenital heart defects; VSD, Ventriculap Septal Defect; ASD, Atrial Septal Defect; TOF, Tetralogy of Fallot; TGA, D-Transposition of the Great Arteries; AVSD, Atrioventricular Septal Defect; TA, truncus arteriosus; LOSD, left-sided obstructive defects. CTA, Conotruncal defects.

a Reported number of cases and control subjects with available exposure information.

b Adjustment variables: A, gestational age; B, birth weight; C, delivery way; D, plurality; E, outcome; F, household registry; G, gestational diabetes mellitus; H, gestational hypertension, I severe preeclampsia; J, residence of mothers; K, maternal age
